# Menstrual knowledge, attitudes and behaviours among secondary school students in Uganda: a longitudinal study

**DOI:** 10.1186/s12889-026-27726-1

**Published:** 2026-06-04

**Authors:** Rodah Nambi, Levicatus Mugenyi, Ratifah Batuusa, Kate A Nelson, Sheila Irene Kisakye, Stephen Lagony, Connie Alezuyo, Belen Torondel, Vishna Shah, Helen A Weiss

**Affiliations:** 1 MRC/UVRI and LSHTM Uganda Research Unit, Entebbe, Uganda; 2 MRC International Statistics and Epidemiology Group, London School of Hygiene & Tropical Medicine, London, UK; 3 Ministry of Education and Sports, Kampala, Uganda; 4 Department of Disease Control, London School of Hygiene and Tropical Medicine, London, UK

**Keywords:** Menstrual health, Menstrual education, Menstrual knowledge and attitudes, Cluster-randomised trial, Adolescents, Uganda

## Abstract

**Background:**

Little is known about the effect of menstrual knowledge, and of menstrual education interventions, on broader dimensions of menstrual health. We assessed factors associated with menstrual knowledge and attitudes, and the association of menstrual knowledge with menstrual health related outcomes among female Ugandan adolescents enrolled in the control arm of a menstrual health intervention trial.

**Methods:**

We conducted a secondary data analysis of longitudinal data from control arm participants in a cluster-randomised trial conducted in Ugandan secondary schools. Endline data were collected one year after baseline. We identified baseline factors associated with the menstrual knowledge and attitudes at endline, and estimated associations of menstrual knowledge items with menstrual practice needs and menstrual confidence at endline, using random-effects Poisson and linear regression analyses to estimate adjusted incidence rate ratios (aIRR) and mean differences (aMD) respectively.

**Results:**

Among 1453 female and 317 male participants in 30 control arm schools who completed both baseline and endline surveys, there was evidence of small associations between better menstrual knowledge at endline with better baseline knowledge (both male and female participants), and in females only, being in a private school (aIRR = 1.05, 95%CI 1.00-1.11), and knowledge about menstruation prior to menarche (aIRR = 1.08, 95%CI 1.03–1.14). Positive attitudes towards menstruation at endline were associated with baseline positive attitudes (male and female participants), and with knowledge prior to menarche (aIRR = 1.10, 95%CI 1.01–1.21). Participants with better knowledge scores had better menstrual experiences (Menstrual Practice Needs Scale: aMD = 0.19, 95%CI 0.08–0.30, *p* < 0.001 for high versus low knowledge scores).

**Conclusions:**

Female adolescents who know about menstruation before menarche have slightly greater menstrual knowledge and positive attitudes towards menstruation. In turn, menstrual knowledge is associated with fewer unmet menstrual practice needs. Future interventions should consider tailoring educational content to address specific knowledge gaps and cultural attitudes and include boys.

**Trial registration number:**

ISRCTN45461276 Registration date: 16/09/2021

**Supplementary Information:**

The online version contains supplementary material available at 10.1186/s12889-026-27726-1.

## Background

Menstrual health is defined as complete physical, mental, and social well-being in relation to the menstrual cycle [1]. Menstrual health is multi-dimensional, including menstrual knowledge (access to accurate, timely, age-appropriate information about the menstrual cycle and menstruation), attitudes (experiencing a positive and respectful environment in relation to the menstrual cycle, free from stigma and psychological distress), self-efficacy (ability to confidently care for their bodies and make informed decisions about self-care throughout their menstrual cycle) and menstrual practices (accessing and using effective and affordable menstrual materials, having supportive facilities, and cleaning and/or disposing of used materials) [1].

Adolescents often have insufficient knowledge to manage menstruation [2–4], exacerbated by prevalent taboos, myths and misconceptions. These cultural constructs contribute to negative perceptions and attitudes towards menstruation within many communities [3–5]. A review of 81 quantitative and qualitative studies from 25 low- and middle-income countries (LMICs) showed that adolescent girls (aged 10–19 years) often have inadequate knowledge about menstruation before they experience it, with approximately half of girls not aware of menstruation prior to menarche [2]. A scoping review of younger adolescents (aged 10–14 years) identified 44 studies across 12 countries and found that many adolescents in LMICs are inadequately prepared for the onset of puberty. They often lack sufficient knowledge about menstruation, only learning about menstruation after experiencing their first period, and this can contribute to negative attitudes towards menstruation [6]. Further, adolescents frequently encounter challenges with managing menstruation, and experiences with menarche are often linked to negative emotions [3]. Gender disparities in menstrual health education, leaves male adolescents less informed about menstruation, contributing to negative attitudes and behaviours [3, 5, 7–9].

Educational interventions delivered by trained personnel can be effective in improving menstrual knowledge and attitudes of young female adolescents (age 10–14 years) [5]. Among 11 studies included in a systematic review, the most effective strategy for improving knowledge was interactive small group or peer-led interventions (average Cohen d = 5.34 and 10.04), rather than pamphlet distribution (d = 0.33) [5].

To date, epidemiological studies on interventions or determinants of menstrual knowledge and attitudes have been relatively small (30–416 participants), and few have studied whether menstrual knowledge and attitudes improve menstrual experiences and confidence. Most previous studies have employed cross-sectional designs with design limitations, and there is a need for longitudinal perspectives to assess temporal effects [2, 3, 5]. To address this evidence gap, we analysed longitudinal data from a school-based menstrual health intervention trial in Uganda [10] conducted from 2021 to 2024, to assess factors associated with menstrual knowledge and attitudes among secondary school adolescents, and the associations of menstrual knowledge with other dimensions of menstrual health. The intervention included puberty education, development of a drama skit, delivery of a menstrual health kit and training, pain management, Water, Sanitation and Hygiene (WASH) improvements, and creation of a Menstrual Health Action Group, while enhanced usual care involved a copy of the Government circular on Provision of Menstrual Hygiene Management for primary and secondary schools, the 2018 National Sexuality Education framework for each school and copies of the Government Menstruation Management Reader distributed to each girl and boy in Senior 2 (S2) [10]. There was strong evidence that the intervention improved multiple menstrual-related outcomes including menstrual knowledge (*p* = 0.0001) and attitudes (*p* < 0.001) scores in female and male students [11].

In this paper, our objectives are (i) to identify baseline factors associated with endline menstrual knowledge and attitudes in female and male control arm participants; and (ii) to assess associations of menstrual knowledge with menstrual practice needs and menstrual confidence in female participants.

## Methods

We used data from the control arm of a cluster-randomized trial evaluating the effect of a multi-component menstrual health intervention (“MENISCUS”) on education, health, and wellbeing among students in 60 secondary schools in Wakiso and Kalungu districts. Wakiso district partly encircles Kampala, the Uganda’s capital city and Kalungu district is approximately 130 km southwest of Kampala and is largely rural.

Details of the trial design and main intervention effects have been published [10, 11]. Secondary schools in the two districts were eligible if they had male and female participants, Senior 1–4 (S1-4) classes, day or mixed day and boarding students, at least minimal WASH facilities [11] and estimated enrolment of 50–125 or 40–125 female S1 students in Wakiso and Kalungu districts respectively. We excluded schools participating in existing menstrual health related programmes and those exclusively for students with disabilities. We assessed all government-aided schools and a random selection of private schools for eligibility using the Government’s 2019 Master List of Education Institutions, phone calls to head teachers, and WASH facility observations. Written school-level consent was sought from the head teacher or director prior to randomisation. We randomised schools 1:1 to receive the MENISCUS intervention or optimised usual care.

All female participants enrolled in S2 and present during the survey period were eligible to be recruited at baseline. Baseline participants were recruited from March 21st to July 5th 2022. At endline, all female participants present and enrolled in S3, based on updated enrolment lists, were eligible to participate in endline assessments (conducted from June 5th to August 22nd 2023). We selected a simple random sample of 15 male S2 participants per school to assess knowledge and attitudes around menstruation. If there were fewer than 15 male participants per school, all were eligible to participate.

For this paper, we analysed data from all female and male participants in the control (enhanced usual care) arm, who completed both baseline and endline surveys. Written informed consent was sought from students aged 18 years and older, and from the parents/guardians provided consent of those who were aged under 18 years, with student assent [10].

The baseline survey was conducted immediately prior to randomisation and the endline survey began one year after randomisation. At each survey, participants completed a self-administered questionnaire on tablets using Open Data Kit (ODK) software. Data were synced daily to an ODK Central Server. Questions included information on socio-demographic characteristics, caregiver characteristics, household-level characteristics, knowledge of puberty and menstruation and attitude towards menstruation. The knowledge and attitudes items are shown in Supplementary Table 1. For the first objective, the outcomes were the number of knowledge and attitude items answered correctly (referred to as the score) at endline, analysed separately by sex. For the second objective, the outcomes were two scale scores reflecting different dimensions of menstrual health among female participants - the Menstrual Practice Needs Scale (MPNS) score [12] and the Self-Efficacy in Addressing Menstrual Needs Scale (SAMNS) score [13]. The MPNS is a 36-item scale with items scored 0–3 to measure the extent to which individuals’ menstrual management practices and environments were perceived to meet their needs during their last menstrual period (LMP). The score was calculated as a weighted mean of participants’ responses to the relevant items (75% weight to the core and school items and 25% weight to the material-specific items). A higher score indicates fewer unmet menstrual needs. The SAMNS is a 26-item scale which measures individuals’ confidence in their capabilities to address their menstrual needs. Participants respond to each item on a scale of 0-100%, representing their confidence in carrying out the specified task. The score is calculated as the mean response to all items. A higher score indicates greater confidence. At baseline and endline, female participants took an educational assessment covering mathematics, science, and English (at endline only), which was developed, administered and marked by the Uganda National Examination Board (UNEB). Menstrual knowledge, attitudes, MPNS and SAMNS were secondary outcomes in the trial.

**Fig. 1:**
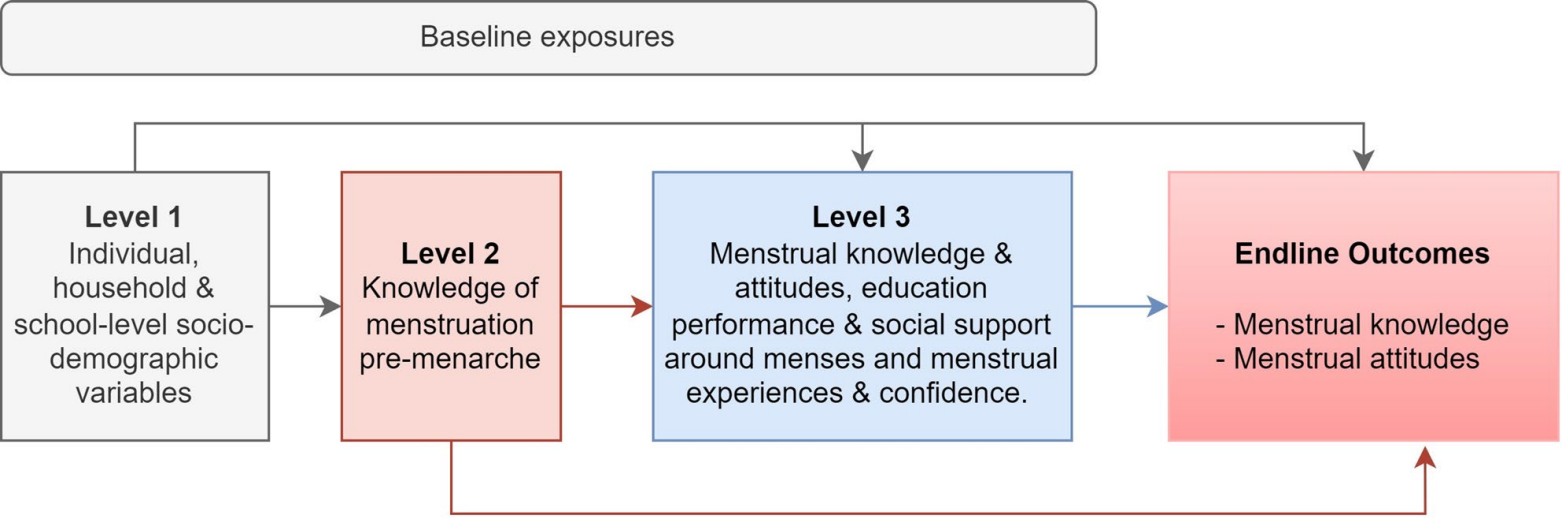
Conceptual framework for endline menstrual knowledge and attitudes among female participants

**Fig. 2:**
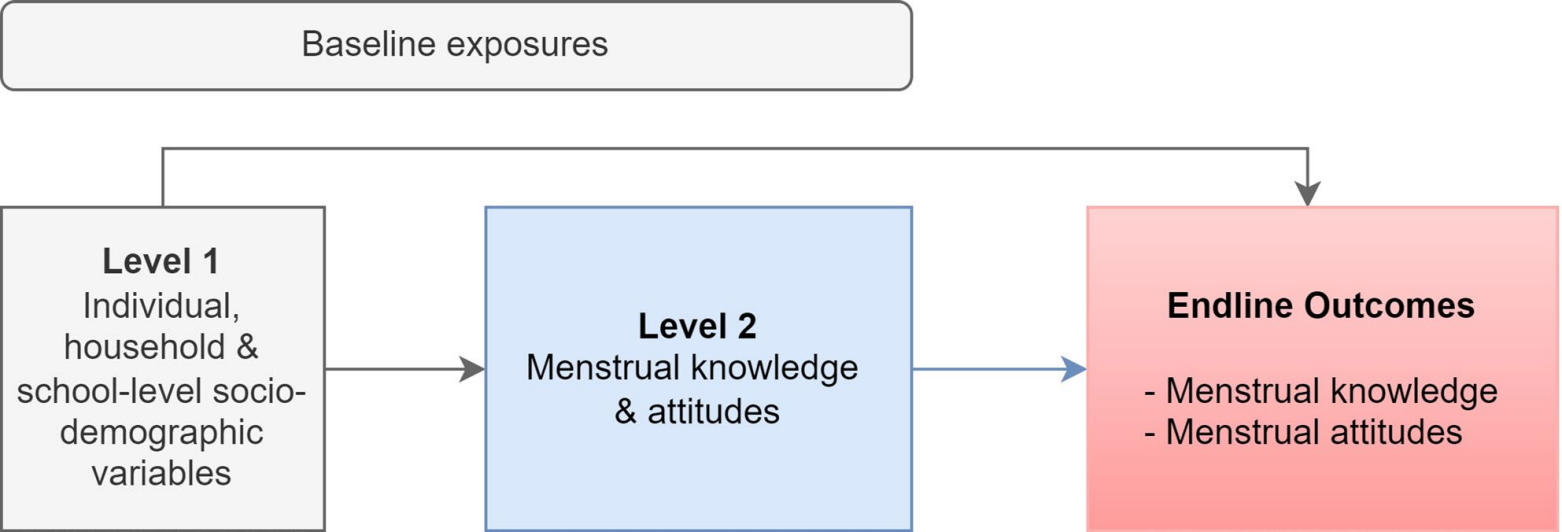
Conceptual framework for endline menstrual knowledge and attitudes among male participants

We conducted analyses using Stata version 18. We used hierarchical conceptual frameworks to assess factors associated with each outcome among female (Fig. 1) and male participants (Fig. 2) [14]. We hypothesized that female participants’ knowledge and attitude towards puberty and menstruation at endline would be influenced by baseline individual, household, and school-level socio-demographic factors (Level 1), knowledge of menstruation pre-menarche (Level 2), baseline menstrual knowledge and attitudes, educational performance, social support around menstruation, and menstrual experiences and confidence (Level 3). Analyses on menstrual experiences and confidence were restricted to participants who reported menstruating in the past six months to minimise recall bias.

We used random-effects Poisson regression to estimate incidence rate ratios (IRRs) and 95% confidence intervals (CIs) for mean knowledge and attitude scores, adjusting for school-level clustering. For females and males, we fitted separate multivariable models for each level in the conceptual framework. Factors at each level were adjusted for each other and for all factors in more distal levels. All factors were retained in subsequent models and the results were presented as adjusted estimates. For example, Level 3 factors were adjusted for each other and for factors at Level 1 and Level 2. P-values were estimated using the global Wald test.

For the second objective, we analysed associations between endline MPNS and SAMNS scores with (i) total knowledge score at endline; (ii) each of the 9 knowledge items at endline; and (iii) reported knowledge of menstruation before menarche at endline. We used random-effects linear regression to estimate adjusted mean differences (aMDs) and 95% CIs for each outcome, adjusting for school-level clustering, baseline MPNS or SAMNS score and knowledge before menarche reported at endline, and baseline variables (Levels 1, 3 and 4). In the final adjusted model, we additionally adjusted each knowledge item for the other 8 knowledge items.

## Results

The baseline trial population comprised 3841 female participants and 874 male participants. Of these, 1921 female and 429 male participants were in control arm schools at baseline, of whom 1453 (75.6%) of female students and 317 (73.9%) of male students completed the endline survey and are included in this paper. The median duration of follow up was 398 days (interquartile range 381–440 days). Female students who were lost to follow-up were more likely to be of no religion (than Christian/Muslim) (*p* = 0.04).

Of the 1453 female participants seen at endline, the mean age at baseline was 15.5 (standard deviation, SD = 0.9) and 790 (54.4%) were day scholars (Table 1). Almost all (*n* = 1404, 96.6%) reported having started menstruating at baseline, of whom 1035 (73.7%) reported knowing about menstruation before menarche. Of the 317 male participants who completed the endline survey, the mean age was 16.0 (SD = 1.1) and 182 (57.4%) were day scholars (Table 1).

**Table 1:** Participants’ characteristics, mean number of knowledge and attitude questions with correct response at endline, and unadjusted and adjusted IRR estimates among female participants

Baseline characteristics	*N* (%)	Knowledge outcome: female participants			Attitude outcome: female participants		
		Knowledge score (out of 9), Mean (SD)	Unadjusted IRR (95% CI)	Adjusted IRR^1^ (95% CI)	Attitude score (out of 3), Mean (SD)	Unadjusted IRR (95% CI)	Adjusted IRR^1^ (95% CI)
N	1,453	5.6 (1.3)			1.9 (0.9)		
Level 1: Individual, household & school-level socio-demographic variables							
Age categories (years)				*P* = 0.19*			*P* = 0.90
<15	142 (9.8%)	5.7 (1.4)	1.00	1.00	1.9 (0.9)	1.00	1.00
15	618 (42.5%)	5.7 (1.3)	1.01 (0.94, 1.09)	1.02 (0.94, 1.10)	1.9 (0.8)	1.02 (0.89, 1.16)	1.00 (0.87, 1.15)
16	524 (36.1%)	5.5 (1.4)	0.97 (0.90, 1.05)	0.99 (0.91, 1.08)	1.8 (0.9)	1.00 (0.87, 1.14)	0.98 (0.85, 1.13)
17	131 (9.0%)	5.5 (1.2)	0.97 (0.88, 1.08)	0.99 (0.89, 1.08)	1.9 (0.8)	1.03 (0.87, 1.23)	1.02 (0.85, 1.22)
18+	38 (2.6%)	4.8 (1.9)	0.86 (0.79, 0.98)	0.87 (0.74, 1.03)	1.8 (1.1)	0.95 (0.73, 1.25)	0.91 (0.69, 1.20)
Ethnicity				*P* = 0.80			*P* = 0.33
Muganda	1,006 (69.2%)	5.6 (1.4)	1.00	1.00	1.8 (0.9)	1.00	1.00
Non Muganda	447 (30.8%)	5.7 (1.3)	1.01 (0.97,1.06)	1.01 (0.96, 1.06)	1.9 (0.9)	1.04 (0.96, 1.12)	1.04 (0.96, 1.14)
Religion			*P* = 0.59	*P* = 0.71		*P* = 0.81	*P* = 0.69
Catholic	470 (32.3%)	5.5 (1.4)	1.00	1.00	1.9 (0.9)	1.00	1.00
Protestant/Born Again/SDA	530 (36.5%)	5.7 (1.4)	1.02 (0.97, 1.08)	1.01 (0.96, 1.07)	1.8 (0.9)	0.98 (0.89, 1.07)	0.96 (0.87, 1.06)
Muslim	446 (30.7%)	5.6 (1.3)	1.01 (0.95,1.07)	1.00 (0.96, 1.06)	1.9 (0.8)	1.01 (0.92, 1.12)	1.01 (0.92, 1.11)
None/Other	7 (0.5%)	6.6 (1.3)	1.18 (0.88,1.59)	1.17 (0.87, 1.57)	2.1 (1.1)	1.14 (0.69, 1.91)	1.12 (0.66, 1.87)
Type of caregiver				*P* = 0.12			*P* = 0.86
Mother	846 (58.2%)	5.5 (1.4)	1.00	1.00	1.9 (0.9)	1.00	1.00
Other	607 (41.8%)	5.7 (1.4)	1.04 (1.00, 1.09)	1.04 (0.99, 1.08)	1.9 (0.9)	1.00 (0.92, 1.08)	0.99 (0.92, 1.07)
Household size				*P* = 0.96			*P* = 0.87
>=8 people	554 (38.1%)	5.6 (1.4)	1.00	1.00	1.9 (0.9)	1.00	1.00
6–8 people	476 (32.8%)	5.6 (1.4)	1.01 (0.96,1.07)	1.01 (0.96, 1.06)	1.9 (0.9)	1.01 (0.93, 1.11)	1.00 (0.92, 1.10)
0–5 people	423 (29.1%)	5.6 (1.3)	1.01 (0.96,1.06)	1.01 (0.95, 1.06)	1.8 (0.9)	0.99 (0.90, 1.09)	0.98 (0.89, 1.08)
Social Economic Status (SES)				*P* = 0.66			*P* = 0.83
Lowest	557 (19.2%)	5.3 (1.5)	1.00	1.00	1.8 (0.9)	1.00	1.00
Medium-low	593 (20.4%)	5.6 (1.3)	1.06 (0.99, 1.14)	1.05 (0.97, 1.13)	1.9 (0.9)	1.03 (0.91, 1.16)	1.03 (0.91, 1.17)
Medium	570 (19.6%)	5.5 (1.3)	1.04 (0.97, 1.12)	1.01 (0.94, 1.09)	1.8 (0.9)	1.00 (0.88, 1.14)	1.02 (0.90, 1.16)
Medium-high	604 (20.8%)	5.7 (1.3)	1.06 (0.99, 1.14)	1.02 (0.94, 1.10)	1.8 (0.9)	0.99 (0.87, 1.12)	1.01 (0.89, 1.15)
Highest	577 (19.9%)	5.9 (1.4)	1.10 (1.02, 1.19)	1.04 (0.97, 1.12)	1.9 (0.9)	1.04 (0.92, 1.18)	1.07 (0.94, 1.22)
Schooling category				*P* = 0.21			*P* = 0.09
Day	790 (54.4%)	5.5 (1.4)	1.00	1.00	1.9 (0.9)	1.00	1.00
Boarding	663 (45.6%)	5.8 (1.3)	1.05 (1.00, 1.10)	1.03 (0.99, 1.08)	1.8 (0.9)	0.93 (0.86, 1.01)	0.93 (0.86, 1.01)
Age at menarche in years				*P* = 0.86			*P* = 0.71
≤12	292 (20.1%)	5.6 (1.4)	1.00	1.00	1.8 (0.9)	1.00	1.00
13	524 (36.1%)	5.7 (1.3)	1.02 (0.96, 1.09)	1.03 (0.96, 1.09)	1.9 (0.9)	1.06 (0.96, 1.18)	1.06 (0.95, 1.18)
14	455 (31.3%)	5.6 (1.4)	1.01 (0.95, 1.08)	1.03 (0.97, 1.10)	1.9 (0.9)	1.05 (0.94, 1.17)	1.05 (0.94, 1.17)
15+	133 (9.2%)	5.3 (1.4)	0.97 (0.89, 1.06)	1.00 (0.92, 1.10)	1.9 (0.9)	1.08 (0.93, 1.25)	1.09 (0.93, 1.27)
Not started menarche	49 (3.4%)	5.6 (1.2)	1.00 (0.88, 1.14)	1.01 (0.89, 1.15)	1.7 (1.0)	0.97 (0.77, 1.22)	0.96 (0.76, 1.21)
District				*P* = 0.003			*P* = 0.36
Kalungu	309 (21.3%)	5.1 (1.4)	1.00	1.00	1.8 (0.9)	1.00	1.00
Wakiso	1,144 (78.7%)	5.7 (1.3)	1.12 (1.06, 1.18)	1.10 (1.03, 1.16)	1.9 (0.9)	1.03 (0.94, 1.14)	1.05 (0.95, 1.16)
School ownership				*P* = 0.06			*P* = 0.08
Government	465 (32.0%)	5.3 (1.4)	1.00	1.00	2.0 (0.9)	1.00	1.00
Private	988 (68.0%)	5.8 (1.3)	1.09 (1.04, 1.14)	1.05 (1.00, 1.11)	1.8 (0.9)	0.93 (0.86, 1.01)	0.92 (0.84, 1.01)
School-level mean UNEB score				*P* = 0.46			*P* = 0.67
Low	781 (53.8%)	5.5 (1.4)	1.00	1.00	1.9 (0.9)	1.00	1.00
High	672 (46.2%)	5.7 (1.3)	1.04 (0.99, 1.10)	1.02 (0.97, 1.07)	1.8 (0.9)	0.98 (0.91, 1.06)	1.02 (0.93, 1.11)
Level 2: Knowledge of menstruation pre-menarche (excluding *N* = 102 who had not started menarche)							
Knowledge of menstruation before menarche				*P* = 0.004			*P* = 0.04
No	369 (25.4%)	5.2 (1.4)	1.00	1.00	1.7 (0.9)	1.00	1.00
Yes	1,035 (71.2%)	5.7 (1.3)	1.09 (1.03, 1.15)	1.08 (1.03, 1.14)	1.9 (0.9)	1.12 (1.01, 1.15)	1.10 (1.01, 1.21)
Level 3: Menstrual knowledge & attitude, educational performance, social support around menses^2^, and menstrual experiences^3^ & confidence^3^ at baseline							
Number of knowledge questions answered correctly				*P* < 0.001*			*P* = 0.31*
Low 0/3	230 (15.8%)	4.6 (1.4)	1.00	1.00	1.7 (1.0)	1.00	1.00
Medium 4/6	1,000 (68.8%)	5.6 (1.2)	1.23 (1.16, 1.32)	1.19 (1.11, 1.23)	1.9 (0.9)	1.12 (1.01, 1.26)	1.08 (0.96, 1.21)
High 7/9	223 (15.3%)	6.5 (1.0)	1.44 (1.33, 1.55)	1.35 (1.24, 1.46)	2.0 (0.8)	1.19 (1.04, 1.36)	1.09 (0.94, 1.26)
Number of attitude questions with positive response				*P* = 0.37*			*P* < 0.001*
0	249 (17.1%)	5.4 (1.4)	1.00	1.00	1.5 (1.0)	1.00	1.00
1	482 (33.2%)	5.5 (1.3)	1.02 (0.96, 1.09)	1.00 (0.93, 1.07)	1.7 (0.9)	1.14 (1.01, 1.30)	1.13 (1.00, 1.29)
2	494 (34.0%)	5.7 (1.3)	1.06 (0.99, 1.13)	1.01 (0.95, 1.08)	2.0 (0.7)	1.40 (1.24, 1.58)	1.36 (1.20, 1.54)
3	228 (15.7%)	5.9 (1.3)	1.10 (1.02, 1.19)	1.03 (0.95, 1.12)	2.4 (0.8)	1.62 (1.42, 1.85)	1.57 (1.37, 1.80)
Individual UNEB score (mean z-score for 3 subjects)				*P* = 0.004			*P* = 0.21
Low (≤-0.3)	757 (52.1%)	5.3 (1.4)	1.00	1.00	1.8 (0.9)	1.00	1.00
High (>-0.3)	696 (47.9%)	6.0 (1.2)	1.13 (1.08, 1.18)	1.07 (1.02, 1.13)	1.9 (0.8)	1.07 (1.00, 1.16)	1.05 (0.97, 1.15)
Social support (someone to ask for advise/help seeking)^1^				*P* = 0.21			
No	225 (16.0%)	5.6 (1.3)	1.00	1.00			
Yes	1,179 (84.0%)	5.7 (1.3)	1.03 (0.96, 1.10)	1.01 (0.95, 1.08)			
No menses in past 6 months	205 (14.2%)	5.2 (1.5)	0.93 (0.87, 0.98)	0.95 (0.86, 1.04)			
Menstrual experience (MPNS score)^3^							*P* = 0.78
Low (0-1.875]	808 (28.0%)				1.8 (0.9)	1.00	1.00
Medium (1.875–2.375)	811 (28.1%)				1.9 (0.9)	1.05 (0.94, 1.16)	1.03 (0.93, 1.15)
High (2.375-3.00]	854 (29.6%)				2.0 (0.8)	1.10 (1.00, 1.22)	1.04 (0.93, 1.17)
No menses in past 6 months	409 (14.1%)				-	-	-
Menstrual confidence (SAMNS score)^3^							*P* = 0.55
Low confidence (0-52.3]	797 (27.5%)				1.8 (0.9)	1.00	1.00
Medium confidence (52.3–69.6]	840 (29.0%)				1.8 (0.9)	0.99 (0.90, 1.10)	0.95 (0.86, 1.06)
High confidence (69.6–100]	855 (29.5%)				2.0 (0.8)	1.08 (0.98, 1.19)	1.00 (0.90, 1.11)
No menses in past 6 months	409 (14.1%)				-	-	-
Worried about the next period^3^							*P* = 0.25
No	1365 (47.1%)				2.0 (0.9)	1.00	1.00
Yes	1127 (38.8%)				1.8 (0.9)	0.91 (0.83, 0.98)	0.95 (0.87, 1.03)
No menses in past 6 months	409 (14.1%)				-	-	-

^1^ Adjusted for variables at the same or more distal levels (Fig. 1); ^2^Only for the knowledge outcome; ^3^Only for the attitude outcome; *P-value based on trend test

For the 9 knowledge items (Supplementary Table 1), the mean score (number correct) was 5.1 (SD = 1.5) at baseline and 5.6 (SD = 1.3) at endline among female participants, and 4.8 (SD = 1.4) at baseline and 5.4 (SD = 1.4) at endline among male participants.

**Fig. 3:**
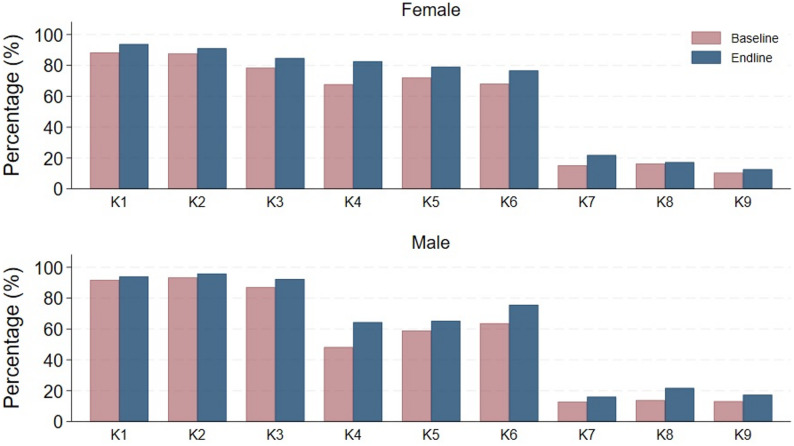
. Percentage of participants with correct knowledge about puberty and menstruation at baseline and endline by sex

There was variability in the proportion of participants correctly answering each question (Fig. 3). The questions with most correct responses (> 80%) among both sexes at both baseline and endline were “K1: What is menstrual period blood” and “K2: The physical changes related to puberty usually start between 10 and 14 years of age in girls, and between 12 and 16 years in boys”. The questions answered least well (< 40% correct) were “K7: When during the menstrual cycle is a woman most likely to become pregnant?”, “K8: What is the entrance to the uterus called?”, and “K9: How many days are there usually between menstrual periods?”.

There were improvements in knowledge between baseline and endline among the control arm participants (Fig. 3), especially for “K4: How long does a menstrual period (bleeding) usually last?” (67.8% to 82.3% in females; 48.3% to 64.4% in males).

**Fig. 4:**
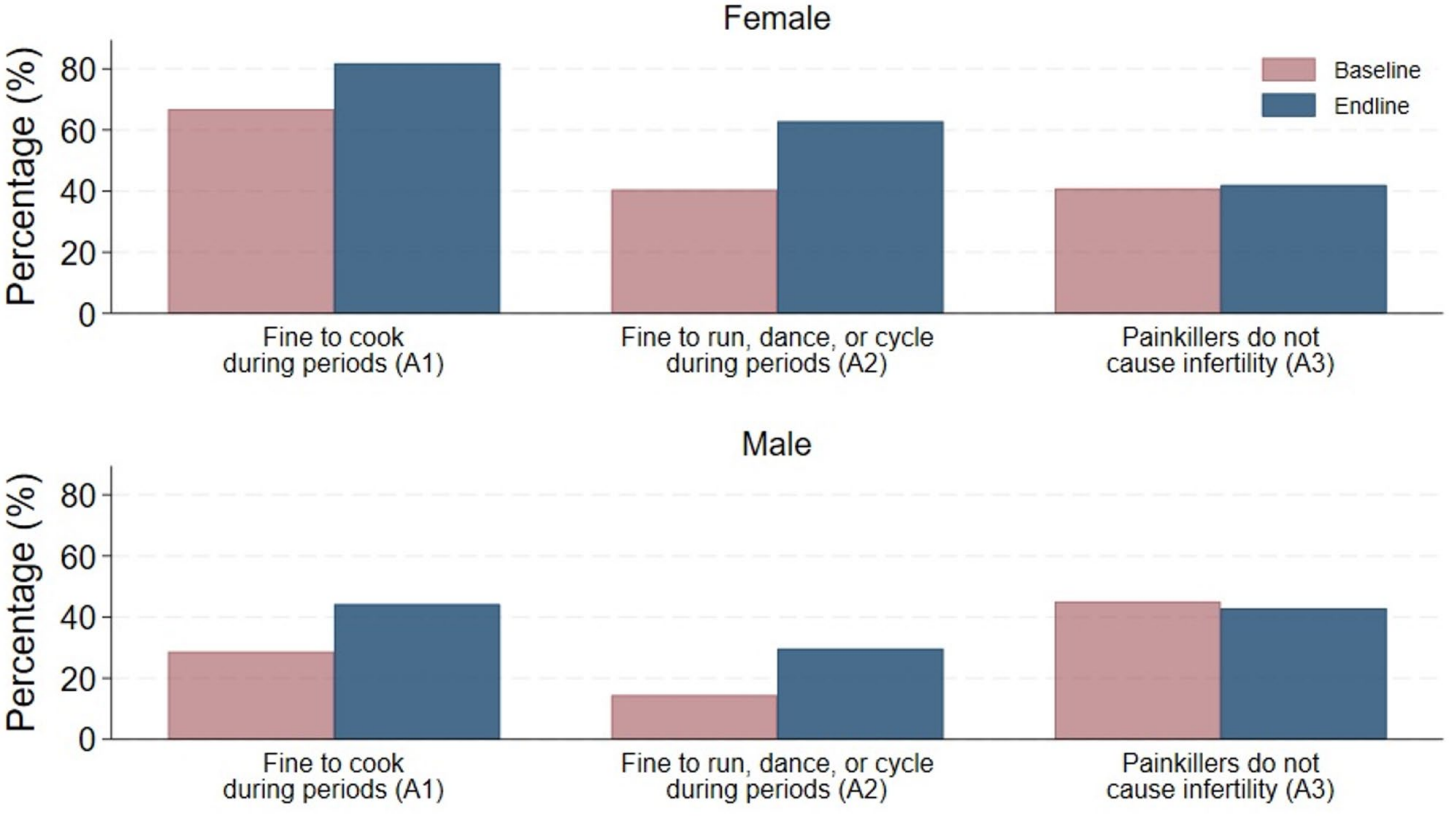
Percentage of participants with positive attitude about puberty and menstruation at baseline and endline, by sex

The mean attitude score (out of 3) increased from 1.5 (SD = 1.0) at baseline to 1.9 (SD = 0.9) at endline among female participants, and from 0.9 (SD = 0.9) at baseline to 1.2 (SD = 1.0) at endline among male participants. There were improvements from baseline to endline for questions A1: “It is fine to cook during periods” (66.8% to 81.8% in females; 28.7% to 44.2% in males, and A2 “It is fine to run, dance or cycle during periods” (40.5% to 62.8% in females; 14.5% to 20.6% in males) (Fig. 4).

### Factors associated with endline knowledge and attitudes

Among female participants, endline knowledge scores were higher among students in Wakiso vs. Kalungu district (mean 5.7 vs. 5.1, aIRR = 1.10, 95%CI 1.03–1.16), those in private schools (mean 5.8 vs. 5.3; aIRR = 1.05, 95%CI 1.00-1.11), and those with knowledge of menstruation before menarche (5.7 vs. 5.2, aIRR = 1.08, 95% CI: 1.03, 1.14) after adjustment for potential confounding variables (Table 1). There was also strong evidence that endline knowledge score was higher among those with higher baseline knowledge scores (mean 6.5 vs. 4.6 for those with 7–9 vs. 0–3 knowledge questions answered correctly at baseline; aIRR = 1.35, 95%CI 1.24–1.46) and higher educational performance using the UNEB score (aIRR = 1.07, 95%CI 1.02–1.13 for high vs. low baseline UNEB score).

Endline attitude scores were higher among females with knowledge of menstruation before menarche (mean 1.9 vs. 1.7, aIRR = 1.10, 95%CI:1.01–1.21), and higher baseline attitude scores (mean 2.4 vs. 1.5 for those with 3 vs. 0 questions correct at baseline; aIRR = 1.57, 95%CI 1.37–1.80). (Table 1).

Endline knowledge in males was higher among males with better baseline knowledge (mean 6.7 vs. 4.5 for those with scores of 7–9 vs. 0–3; aIRR = 1.41, 95%CI 1.16–1.71). There was no evidence that other exposures were associated with endline knowledge (Table 2). Similarly, there was strong evidence that endline attitudes among male participants were associated with baseline attitudes (mean scores 2.5 vs. 0.8 for those with 3 vs. 0 baseline questions correct, aIRR = 2.94, 95%CI 2.03–4.27) (Table 2).

**Table 2:** Participants’ characteristics, mean number of knowledge and attitude questions with correct response at endline, and unadjusted and adjusted IRR estimates among male participants

Baseline characteristics	*N* (%)	Knowledge outcome: male participants			Attitude outcome: male participants		
		Knowledge score, Mean (SD) Max: 9	Unadjusted IRR (95% CI)	Adjusted IRR^1^ (95% CI)	Attitude score Mean (SD) Max 3	Unadjusted IRR (95% CI)	Adjusted IRR^1^ (95% CI)
N	317	5.4 (1.4)			1.2 (1.0)		
Level 1: Individual, household & school-level socio-demographic variables							
Age categories (years)				*P* = 0.91			*P* = 0.08
<15	25 (7.9%)	5.6 (1.5)	1.00	1.00	1.7 (1.1)	1.00	1.00
15	89 (28.1%)	5.4 (1.5)	0.96 (0.80, 1.16)	0.96 (0.79, 1.16)	1.0 (1.0)	0.59 (0.41, 0.85)	0.59 (0.41, 0.86)
16	97 (30.6%)	5.5 (1.4)	0.99 (0.82, 1.19)	1.02 (0.84, 1.23)	1.1 (1.0)	0.66 (0.46, 0.94)	0.67 (0.46, 0.96)
17	79 (24.9%)	5.4 (1.3)	0.97 (0.80, 1.17)	0.99 (0.81, 1.20)	1.2 (1.0)	0.71 (0.49, 1.01)	0.72 (0.50, 1.04)
18+	27 (8.5%)	5.0 (1.7)	0.90 (0.71, 1.14)	0.95 (0.75, 1.22)	1.1 (1.0)	0.65 (0.41, 1.03)	0.63 (0.38, 1.03)
Ethnicity				*P* = 0.51			*P* = 0.39
Muganda	225 (71.0%)	5.5 (1.4)	1.00	1.00	1.2 (1.0)	1.00	1.00
Non Muganda	92 (29.0%)	5.3 (1.4)	0.98 (0.88,1.09)	0.96 (0.86, 1.07)	1.2 (1.0)	1.01 (0.81, 1.26)	0.91 (0.71, 1.15)
Religion				*P* = 0.66			*P* = 0.19
Catholic	118 (37.2%)	5.4 (1.5)	1.00	1.00	1.0 (1.0)	1.00	1.00
Protestant/Born Again/SDA	113 (35.6%)	5.3 (1.3)	0.98 (0.87, 1.09)	0.98 (0.88, 1.11)	1.2 (1.0)	1.21 (0.95, 1.54)	1.25 (0.97, 1.61)
Muslim	85 (26.8%)	5.8 (1.4)	1.07 (0.95,1.20)	1.05 (0.93,1.19)	1.3 (1.0)	1.26 (0.97, 1.63)	1.24 (0.95, 1.61)
None/Other	1 (0.3%)	4.0 (.)	0.74 (0.28,1.99)	0.72 (0.26,1.97)	2.0 (.)	1.97 (0.49, 7.96)	2.38 (0.54, 10.51)
Type of caregiver				*P* = 0.45			*P* = 0.46
Mother	160 (50.5%)	5.6 (1.4)	1.00	1.00	1.1 (1.0)	1.00	1.00
Other	157 (49.5%)	5.3 (1.5)	0.94 (0.86, 1.04)	0.96 (0.87, 1.06)	1.2 (1.0)	1.03 (0.84, 1.26)	1.08 (0.87, 1.33)
Household size				*P* = 0.50			*P* = 0.30
* ≥* 8 people	120 (37.9%)	5.4 (1.5)	1.00	1.00	1.2 (1.0)	1.00	1.00
6–7 people	101 (31.9%)	5.3 (1.4)	0.97 (0.87,1.09)	0.98 (0.87, 1.10)	1.0 (1.0)	0.86 (0.67, 1.10)	0.84 (0.65, 1.09)
<5 people	96 (30.3%)	5.6 (1.4)	1.04 (0.92,1.16)	1.05 (0.93, 1.18	1.2 (1.0)	0.99 (0.78, 1.27)	1.01 (0.79, 1.30)
Social Economic Status (SES)				*P* = 0.73			*P* = 0.46
Lowest	52 (16.4%)	5.1 (1.7)	1.00	1.00	1.2 (1.1)	1.00	1.00
Medium-low	62 (19.6%)	5.4 (1.3)	1.06 (0.90, 1.24)	1.04 (0.88, 1.23)	1.2 (0.9)	1.03 (0.73, 1.43)	1.02 (0.73, 1.44)
Medium	69 (21.8%)	5.2 (1.4)	1.02 (0.87, 1.20)	1.01 (0.86, 1.19)	1.0 (1.0)	0.86 (0.61, 1.21)	0.84 (0.60, 1.19)
Medium-high	65 (20.5%)	5.8 (1.3)	1.13 (0.97, 1.33)	1.11 (0.94, 1.30)	1.3 (1.0)	1.07 (0.77, 1.48)	1.02 (0.73, 1.43)
Highest	69 (21.8%)	5.7 (1.3)	1.12 (0.95, 1.30)	1.07 (0.91, 1.27)	1.1 (0.9)	0.89 (0.63, 1.24)	0.81 (0.56, 1.16)
Schooling category				*P* = 0.21			*P* = 0.15
Day	182 (57.4%)	5.3 (1.4)	1.00	1.00	1.1 (1.0)	1.00	1.00
Boarding	135 (42.6%)	5.7 (1.4)	1.08 (0.98, 1.18)	1.07 (0.96, 1.19)	1.2 (1.0)	1.11 (0.90, 1.36)	1.19 (0.94, 1.50)
District				*P* = 0.38			*P* = 0.71
Kalungu	89 (28.1%)	5.2 (1.6)	1.00	1.00	1.1 (1.0)	1.00	1.00
Wakiso	228 (71.9%)	5.5 (1.3)	1.07 (0.96, 1.19)	1.05 (0.94, 1.18)	1.2 (1.0)	1.03 (0.83, 1.31)	1.05 (0.82, 1.34)
School ownership				*P* = 0.86			*P* = 0.64
Government	96 (30.3%)	5.2 (1.5)	1.00	1.00	1.1 (1.0)	1.00	1.00
Private	221 (69.7%)	5.5 (1.4)	1.05 (0.95, 1.17)	1.01 (0.90, 1.13)	1.2 (1.0)	1.03 (0.82, 1.28)	0.94 (0.74, 1.20)
Level 2: Menstrual knowledge & attitudes at baseline							
Number of knowledge questions answered correctly				*P* < 0.001*			*P* = 0.73
Low 0/3	55 (17.4%)	4.5 (1.4)	1.00	1.00	1.1 (1.0)	1.00	1.00
Medium 4/6	231 (72.9%)	5.5 (1.3)	1.22 (1.06, 1.40)	1.20 (1.04, 1,37)	1.2 (1.0)	1.11 (0.84, 1.48)	0.99 (0.73, 1.32)
High 7/9	31 (9.8%)	6.7 (1.2)	1.50 (1.25, 1.80)	1.41 (1.16, 1.71)	1.3 (0.9)	1.25 (0.84, 1.87)	1.13 (0.74, 1.73)
Number of attitude questions with positive response				*P* = 0.28*			*P* < 0.001*
0	126 (39.7%)	5.4 (1.4)	1.00	1.00	0.8 (0.8)	1.00	1.00
1	122 (38.5%)	5.2 (1.4)	0.97 (0.87, 1.08)	0.97 (0.86, 1.09)	1.1 (1.0)	1.42 (1.10, 1.84)	1.43 (1.10, 1.88)
2	49 (15.5%)	5.6 (1.5)	1.03 (0.89, 1.18)	1.01 (0.88, 1.17)	1.8 (1.0)	2.23 (1.67, 2.98)	2.28 (1.68, 3.09)
3	20 (6.3%)	6.5 (1.2)	1.20 (1.00, 1.45)	1.17 (0.96, 1.43)	2.5 (0.6)	3.12 (2.21, 4.39)	2.94 (2.03, 4.27)

^1^ Adjusted for variables at the same or more distal levels (Fig. 2); *P-value based on trend test

### Associations of menstrual knowledge with menstrual health practice needs and menstrual confidence

Of the 1453 female participants seen at endline, 1360 (93.6%) had menstruated in the past 6 months and were included in analyses of associations of MPNS and SAMNS scales with endline menstrual knowledge (Table 3). Data on endline MPNS were missing for 3 participants (*N* = 1357). Overall, the mean MPNS score was 2.3 (SD = 0.5), with a maximum score of 3.0 representing no unmet needs. The mean SAMNS score was 64.2 (SD = 18.1) out of a maximum possible confidence score of 100. There was strong evidence that MPNS scores were higher among those with better menstrual knowledge scores, after adjustment for baseline knowledge scores and potential confounders. There was strong evidence that MPNS and SAMNS scores were both associated with knowledge of menstruation before menarche. MPNS scores were associated with correct answers to specific knowledge questions K5 (lack of menstruation during pregnancy) and K6 (age when women usually stop menstruating), adjusted for other knowledge items and potential confounders. The absolute difference was small (MPNS: aMD = 0.19, 95%CI 0.08–0.30 (standardised mean difference = 0.37) for those in the highest vs. lowest baseline knowledge category).

**Table 3:** Knowledge items, mean MPNS score and SAMNS score at endline, and unadjusted and adjusted mean difference (MD) estimates among female participants menstruating within past 6 months at endline, adjusted for baseline factors

Endline knowledge items (* denotes correct response)	Endline Menstrual Experience (MPNS Score; scale 0–3)^1^				Endline Menstrual Confidence (SAMNS Score; scale 0-100)			
	*N* (%)	Mean (SD)	Adjusted MD^2^ (95 CI)	Additionally adjusted MD (95 CI) ^3^	*N* (%)	Mean (SD)	Adjusted MD^4^ (95 CI)	Additionally adjusted MD (95 CI) ^5^
N	1357	2.3 (0.5)			1360	64.2 (18.1)		
Number of knowledge questions answered correctly			P-trend = 0.006				P-trend = 0.25	
Low 0/3	86 (6.3%)	2.1 (0.5)	0	n/a	86 (6.3%)	59.6 (17.8)	0	n/a
Medium 4/6	935 (68.9%)	2.3 (0.5)	0.16 (0.06, 0.25)	n/a	938 (69.0%)	63.9 (18.2)	1.80 (-2.04, 5.66)	n/a
High 7/9	336 (24.8%)	2.4 (0.5)	0.19 (0.08, 0.30)	n/a	336 (24.7%)	66.2 (17.7)	2.66 (-1.65, 6.98)	n/a
K1: What is menstrual period blood?			*P* = 0.76	*P* = 0.87			*P* = 0.76	*P* = 0.30
Blood from the lining of the uterus (womb)*	1,277 (94.1%)	2.3 (0.5)	0	0	1,280 (94.1%)	64.3 (18.2)	0	0
Blood from the stomach	35 (2.6%)	2.3 (0.5)	0.02 (-0.12, 0.17)	0.01 (-0.14, 0.15)	35 (2.6%)	62.7 (16.5)	-0.99 (-6.99, 4.99)	1.55 (-4.16, 7.27)
Don’t know	45 (3.3%)	2.2 (0.5)	-0.04 (-0.17, 0.09)	-0.03 (-0.17, 0.10)	45 (3.3%)	63.2 (17.8)	1.75 (-3.50, 7.00)	4.00 (-1.25, 9.25)
K2: The physical changes related to puberty usually start between 10 and 14 years of age in girls, and between 12 and 16 in boys			*P* = 0.85	*P* = 0.94			*P* = 0.30	*P* = 0.63
True*	1,239 (91.3%)	2.3 (0.5)	0	0	1,242 (91.3%)	64.6 (17.9)	0	0
False	44 (3.2%)	2.2 (0.5)	0.02 (-0.11, 0.15)	0.02 (-0.11, 0.16)	44 (3.2%)	61.1 (20.1)	-0.42 (-5.83, 4.98)	-1.45 (-6.54, 3.64)
Don’t know	74 (5.5%)	2.2 (0.5)	-0.02 (-0.13, 0.08)	0.00 (-0.10, 0.11)	74 (5.4%)	60.0 (20.1)	-3.24 (-7.35, 0.87)	-1.65 (-5.70, 2.40)
K3: Changes in the body during puberty happen because of hormones			*P* = 0.84	*P* = 0.91			*P* = 0.15	*P* = 0.25
True*	1,161 (85.6%)	2.3 (0.5)	0	0	1,164 (85.6%)	64.8 (17.9)	0	0
False	62 (4.6%)	2.2 (0.6)	-0.01 (-0.12, 0.10)	-0.02 (-0.13, 0.09)	62 (4.6%)	60.8 (22.4)	-0.86 (-5.37, 3.65)	-1.95 (-6.32, 2.42)
Don’t know	134 (9.9%)	2.2 (0.5)	-0.02 (-0.10, 0.06)	-0.01 (-0.09, 0.07)	134 (9.9%)	60.4 (17.5)	-3.16 (-6.35, 0.03)	-2.41 (-5.54, 0.72)
K4: How long does a menstrual period (bleeding) usually last?			*P* = 0.16	*P* = 0.28			*P* = 0.34	*P* = 0.78
3 to 7 days*	1,131 (83.3%)	2.3 (0.5)	0	0	1,134 (83.4%)	64.4 (17.8)	0	0
Exactly 5 days	194 (14.3%)	2.3 (0.5)	0.01 (-0.06, 0.08)	0.01 (-0.05, 0.08)	194 (14.3%)	64.0 (19.3)	0.33 (-2.38, 3.05)	0.03 (-2.58, 2.64)
About 28 days	12 (0.9%)	2.3 (0.4)	0.06 (-0.19, 0.31)	0.04 (-0.21, 0.30)	12 (0.9%)	65.1 (23.0)	-0.41 (-10.57, 9.76)	-1.86 (-11.46, 7.74)
I don’t know	20 (1.5%)	2.0 (0.5)	-0.20 (-0.39, -0.02)	-0.18 (-0.36, 0.01)	20 (1.5%)	55.9 (19.6)	-6.95 (-14.52, 0.62)	-3.72 (-11.25, 3.80)
K5: Monthly menstruation continues during pregnancy			*P* = 0.005	*P* = 0.006			*P* = 0.14	*P* = 0.26
False*	1,089 (80.3%)	2.3 (0.5)	0	0	1,091 (80.2%)	64.9 (18.1)	0	0
True	114 (8.4%)	2.1 (0.5)	-0.13 (-0.21, -0.05)	-0.14 (-0.22, -0.05)	115 (8.5%)	61.5 (18.1)	-2.87 (-6.29, 0.54)	-2.50 (-5.79, 0.79)
Don’t know	154 (11.3%)	2.2 (0.5)	-0.06 (-0.13, 0.02)	-0.03 (-0.10, 0.06)	154 (11.3%)	61.5 (17.6)	-2.04 (-5.06, 0.98)	-1.32 (-4.30, 1.66)
K6: Usually, women stop menstruating after about age 40–50 years			*P* = 0.004	*P* = 0.007			*P* = 0.25	*P* = 0.50
True*	1,050 (77.4%)	2.3 (0.5)	0	0	1,052 (77.4%)	64.8 (18.1)	0	0
False	92 (6.8%)	2.3 (0.5)	0.03 (-0.06, 0.12)	0.03 (-0.06, 0.12)	92 (6.8%)	62.2 (17.9)	-1.91 (-5.68, 1.86)	-1.68 (-5.28, 1.91)
Don’t know	215 (15.8%)	2.2 (0.5)	-0.10 (-0.17, -0.04)	-0.10 (-0.17, -0.04)	216 (15.9%)	62.0 (18.0)	-1.96 (-4.60, 0.67)	-1.10 (-3.68, 1.47)
K7: When during the menstrual cycle is a woman most likely to become pregnant?			*P* = 0.78	*P* = 0.94			*P* = 0.60	*P* = 0.71
After ovulation*	309 (22.8%)	2.3 (0.5)	0	0	309 (22.7%)	65.5 (17.2)	0	0
Just before her period	393 (29.0%)	2.3 (0.5)	-0.03 (-0.09, 0.04)	-0.02 (-0.09, 0.04)	394 (29.0%)	65.0 (17.9)	-0.17 (-2.83, 2.50)	0.31 (-2.23, 2.85)
During her period	123 (9.1%)	2.3 (0.5)	-0.01 (-0.10, 0.08)	0.00 (-0.09, 0.09)	123 (9.0%)	64.6 (19.1)	-0.08 (-3.83, 3.66)	-0.20 (-3.77, 3.38)
Right after her period	303 (22.3%)	2.3 (0.5)	-0.02 (-0.09, 0.05)	-0.01 (-0.08, 0.06)	305 (22.4%)	63.1 (19.1)	-1.09 (-3.91, 1.73)	-1.47 (-4.15, 1.22)
Don’t know	229 (16.9%)	2.3 (0.5)	-0.05 (-0.12, 0.03)	-0.02 (-0.10, 0.05)	229 (16.8%)	62.4 (17.6)	-2.19 (-5.25, 0.88)	-0.58 (-3.59, 2.43)
K8: What is the entrance to the uterus called?			*P* = 0.97	*P* = 0.99			*P* = 0.45	*P* = 0.23
Cervix*	238 (17.5%)	2.3 (0.5)	0	0	238 (17.5%)	64.8 (18.5)	0	0
Vagina	750 (55.3%)	2.3 (0.5)	-0.00 (-0.07, 0.06)	0.01 (-0.05, 0.07)	752 (55.3%)	64.9 (18.0)	0.82 (-1.74, 3.39)	1.13 (-1.34, 3.59)
Vulva	83 (6.1%)	2.3 (0.5)	-0.02 (-0.13, 0.09)	0.00 (-0.11, 0.10)	83 (6.1%)	66.7 (16.0)	2.81 (-1.56, 7.19)	2.93 (-1.31, 7.16)
Ovary	73 (5.4%)	2.2 (0.5)	-0.03 (-0.14, 0.09)	-0.02 (-0.13, 0.10)	73 (5.4%)	60.8 (17.3)	-0.99 (-5.67, 3.69)	-1.16 (-5.62, 3.30)
Don’t know	213 (15.7%)	2.2 (0.5)	-0.02 (-0.10, 0.06)	0.00 (-0.08, 0.09)	214 (15.7%)	61.4 (18.6)	-0.85 (-4.12, 2.42)	-1.20 (-4.44, 2.05)
K9: How many days are there usually between menstrual periods?			*P* = 0.34	*P* = 0.43			*P* = 0.29	*P* = 0.13
21–45 days*	177 (13.0%)	2.3 (0.5)	0	0	177 (13.0%)	62.7 (17.6)	0	0
Exactly 28 days	519 (38.2%)	2.3 (0.5)	0.00 (-0.07, 0.07)	0.00 (-0.08, 0.07)	519 (38.2%)	65.4 (17.7)	2.91 (-0.81, 5.50)	2.57 (-0.32, 5.46)
Exactly 7 days	402 (29.6%)	2.2 (0.5)	-0.05 (-0.13, 0.03)	-0.04 (-0.10, 0.01)	404 (29.7%)	63.4 (19.2)	2.34 (-0.06, 5.88)	1.11 (-1.92, 4.13)
Don’t know	259 (19.1%)	2.3 (0.5)	-0.02 (-0.10, 0.07)	0.01 (-0.08, 0.09)	260 (19.1%)	64.0 (17.5)	2.11 (-1.23, 5.45)	3.30 (0.01, 6.59)
Knowledge of menstruation before menarche^6^			*P* < 0.001	n/a			*P* < 0.001	n/a
Yes	1,015 (74.8%)	2.3 (0.5)	0	n/a	1,017 (74.8%)	65.5 (17.3)	0	n/a
No	342 (25.2%)	2.1 (0.5)	-0.13 (-0.18, -0.07)	n/a	343 (25.2%)	60.2 (19.8)	-5.32 (-7.54, -3.09)	n/a

* denotes correct response

^1^ 5 participants had missing data on MPNS score and these were excluded from the analysis

^2^ Adjusted for baseline MPNS score, knowledge before menarche (reported at endline), baseline socio-demographic (Level 1), and menstrual-related factors (Level 3)

^3^ Adjusted for factors in footnote 2, and in addition individual knowledge items adjusted for the other knowledge items

^4^ Adjusted for baseline SAMNS score, knowledge before menarche (reported at endline), baseline socio-demographic (Level 1), and menstrual-related factors (Level 4)

^5^ Adjusted for factors in footnote 4, and in addition individual knowledge items adjusted for the other knowledge items

^6^ Knowledge of menstruation before menarche is adjusted for Level 1 (socio-demographic) factors only to avoid adjusting for variables on the causal pathway to the outcomes

There was no evidence of an association of MPNS and SAMNS with other specific knowledge items once other knowledge items were adjusted for.

## Discussion

To our knowledge, this is the first large-scale school-based longitudinal study in sub-Saharan Africa to estimate factors associated with menstrual knowledge and attitudes among male as well as female adolescents, and to relate menstrual knowledge and attitudes to validated measures of broader dimensions of menstrual health. In these control arm participants, we found strong evidence of small associations between knowledge of menstruation before menarche with better menstrual knowledge among female and male adolescents, and with positive attitudes towards menstruation among female adolescents. Menstrual knowledge was associated with small improvements in menstrual experiences, assessed by the menstrual practice needs scales.

A key factor to influence both menstrual knowledge and attitudes in this study was knowing about menstruation prior to menarche. This early awareness may have contributed to a sense of preparedness and facilitated positive associations with menstruation, mitigating feelings of shame [15]. Further, early communication may have encouraged students to engage in discussions and seek additional information, and may suggest the presence of good support networks (parents, teachers, and peers), who play a crucial role in providing information about menstruation and normalising it [2, 3, 16].

Among male participants, better knowledge and positive attitudes towards menstruation at baseline were associated with better endline knowledge and attitudes, respectively. This is likely to reflect retention of the baseline knowledge and attitudes. Similarly, our earlier finding of an effect of the menstrual health intervention on menstrual attitudes among boys (as well as girls) [11] supports evidence from studies that emphasize the need to educate boys about menstruation, with boys expressing interest in learning about menstrual health [9, 17, 18]. A recent pilot study in Bangladesh on engaging boys in menstrual hygiene management interventions highlighted the importance of male participation and involvement as a way to destigmatize menstruation [19]. This could be a key step in breaking taboos and stigmas.

The improvements in menstrual knowledge and attitudes between baseline and endline could be attributed to participants being a year older at endline and gaining knowledge over time, as well as the menstrual health government readers that were distributed in control (and intervention schools) at baseline. There were also some menstrual health activities (e.g. pad delivery) in some of the control schools. Participation in stakeholder workshops and the baseline survey may also have increased awareness of menstrual health.

The slightly poorer endline knowledge and attitudes among older students may be because all participants were in the S3 class cohort at endline, and students who are older in this cohort are likely to have repeated classes due to poor academic performance or missing school and being generally disadvantaged.

We found that female adolescents who were well-informed about menstruation managed their menses somewhat more effectively. While greater menstrual knowledge was associated with improved experiences, the relatively small effect size suggests that addressing structural, social, and material constraints remains essential. These findings align with a systematic review of qualitative studies which found that females who were better informed about menstruation were more confident in managing their periods and a lack of knowledge can lead to confusion, distress, and adherence to inaccurate cultural taboos, which negatively impacted confidence [15]. The review also emphasizes that poor menstrual experiences can negatively impact health and social participation.

We found no evidence that specific types of knowledge questions (e.g. practical knowledge on duration of the menstrual cycle or bleeding versus physiological knowledge versus) were associated with improved menstrual practices and confidence, although some specific questions were associated with these outcomes (lack of menstruation during pregnancy and age when women usually stop menstruating).

Our earlier findings of a positive, though small, effect of the MENISCUS intervention on knowledge and attitudes in female participants and on attitudes in boys aligns with other studies, which have reported improved menstrual knowledge and attitudes in intervention compared to control groups [5, 20, 21]. These studies demonstrate that education sessions, print materials, videos, didactic teaching, menstrual health management training with practical skills, peer education and small group teaching can improve menstrual knowledge. A systematic review [5] found that the effect size on knowledge among those that distributed pamphlets and books was lowest (0.33) in a randomised controlled trial (RCT) among female adolescents in Iran [22], and highest (4.81) in a non-randomised study of lectures followed by discussion, also in Iran [23].

Strengths of our study include the longitudinal design, which allowed us to track how menstrual-related knowledge, attitudes and related factors influence changes in menstrual health at the start and end of a 12-month period, and the inclusion of male as well as female students. The longitudinal design means that exposures were measured before outcomes, allowing us to rule out reverse causality. The high response rate and representative, heterogeneous sample, with participants from rural and urban districts, day and boarding schools and both government-aided and private schools, enhances the generalizability of the findings within this setting. Limitations include that reliance on self-reported data may introduce social desirability bias for menstrual attitudes, where participants might over-report positive attitudes, and the relatively small number of male participants. To minimize social desirability bias, participants self-completed surveys on tablet computers to enhance privacy. The limited items on attitudes and knowledge may not capture all dimensions of participants’ understandings of puberty and menstruation. The study was conducted in two districts in central Uganda, thus the findings may not be generalizable to other regions with different socio-cultural contexts.

## Conclusions

In this Ugandan in-school population, increased knowledge was associated with small increases in menstrual practice needs. Future interventions should consider starting menstrual education before menarche and tailoring educational content to address specific knowledge gaps and cultural attitudes and include boys.

## Supplementary Information

Supplementary Material 1.

## Data Availability

The datasets generated and analysed during the current study are available on request from the London School of Hygiene & Tropical Medicine Data Compass at https://doi.org/10.17037/DATA.00003822, along with the codebook, informed consent documents, and qualitative interview guides. Participants gave informed consent for their data to be published after de-identification.

## Abbreviations

aIRR: Adjusted Incidence Rate Ratio
aMD: Adjusted Mean Difference
CI: Confidence Interval
LMICs: Low- and Middle-Income countries
LMP: Last Menstrual Period
LSHTM: London School of Hygiene and Tropical Medicine
MENISCUS: Menstrual Health Interventions Schooling and Mental Health Symptoms among Ugandan Students
MPNS: Menstrual Practice Needs Scale
NIHR: National Institute of Health Research
ODK: Open Data Kit
RCT: Randomised Control Trial
SAMNS: Self-Efficacy in Addressing Menstrual Needs Scale
SD: Standard Deviation
S2: Senior 2
UNCST: Uganda National Council of Science and Technology
UNEB: Uganda National Examination Board
UVRI-REC: Uganda Virus Research Institute Research and Ethics Committee
WASH: Water, Sanitation, Hygiene
