# Supplementary material for: Menstrual knowledge, attitudes and behaviours among secondary school students in Uganda: a longitudinal study
Source: BMC Public Health. 2026 Jun 4;26:2310. doi: 10.1186/s12889-026-27726-1 (PMC13455388; doi:10.1186/s12889-026-27726-1)
Supplement: Supplementary file 1 [file 12889_2026_27726_MOESM1_ESM.docx]

**Supplementary Table 1: MENISCUS knowledge and attitude questions about puberty and menstruation**

| **Knowledge questions** | **Response options (correct response in bold)** |
| --- | --- |
| K1: What is menstrual period blood? | - Blood from the stomach - **Blood from the lining of the uterus (womb)** - Don't know |
| K2: The physical changes related to puberty usually start between 10 and 14 years of age in girls, and between 12 and 16 in boys. | - **True** - False - Don't know |
| K3: Changes in the body during puberty happen because of hormones | - **True** - False - Don't know |
| K4: How long does a menstrual period (bleeding) usually last? | - **Between 3 to 7 days** - Exactly 5 days - About 28 days - Don't know |
| K5: Monthly menstruation continues during pregnancy | - True - **False** - Don't know |
| K6: Usually, women stop menstruating after about age 40-50 years | - **True** - False - Don't know |
| K7: When during the menstrual cycle is a woman most likely to become pregnant? | - Just before her period - During her period - Right after her period - **After ovulation** - Don’t know |
| K8: What is the entrance to the uterus called? | - Vagina - Vulva - **Cervix** - Ovary - Don’t know |
| K9: How many days are there usually between menstrual periods? | - Exactly 7 days - Exactly 28 days - **Between 21-45 days** - Don’t know |
| **Attitude questions** |  |
| A1: It is fine for a girl to cook during her period | - Disagree a lot - Disagree - Neither agree nor disagree - **Agree** - **Agree a lot** |
| A2: It is fine for a girl to run, dance or cycle during her period |  |
| A3: Painkillers cause problems having children (barrenness) | - **Disagree a lot** - **Disagree** - Neither agree nor disagree - Agree - Agree a lot |
